# Dimensional changes of upper airway after slow vs rapid miniscrew-supported maxillary expansion in adolescents: a cone-beam computed tomography study

**DOI:** 10.1186/s12903-022-02581-9

**Published:** 2022-11-24

**Authors:** Yomna M. Yacout, Nadia M. El-Harouni, Ahmed M. Madian

**Affiliations:** Department of Orthodontics, Faculty of Dentistry, Alexandria University, P. O. Box: 21521, Alexandria, Egypt

**Keywords:** Slow maxillary expansion, Rapid maxillary expansion, Miniscrews, Retropalatal airway, Retroglossal airway

## Abstract

**Background:**

To date, the effects of different activation rates of miniscrew-supported expanders on the airway have not been compared. Hence, the purpose of this retrospective study was to evaluate and compare the effects of slow and rapid miniscrew-supported maxillary expansion on the upper airway dimensions using cone-beam computed tomography (CBCT).

**Methods:**

Data of 20 patients (Age 12 to 16 years old) treated using miniscrew-supported expanders at the Faculty of Dentistry, Alexandria University was collected. The patients were equally divided into two groups according to the activation protocol; slow maxillary expansion (SME): activation once every other day, and rapid maxillary expansion (RME): activation twice daily. CBCT scans obtained pre-expansion and 5 months post-expansion were used to evaluate the changes in the upper airway dimensions. Comparisons between the two time points within each group were done using paired samples t-test. SME and RME groups were compared using independent samples t-test. Significance level was set at *p* < 0.05.

**Results:**

Both groups showed a significant increase in anterior, middle, and posterior nasal cavity width. SME resulted in significantly greater increase of the anterior nasal cavity width than RME (Mean difference between the groups, 2.64 mm; 95% CI, 0.83, 4.45; *p* = 0.007). The dimensions of the retropalatal and retroglossal airways did not change significantly in either group. Both groups resulted in a significant increase of maxillary width, palatal width, and inter-molar width. RME showed a significantly larger increase of inter-molar width than SME (Mean difference between the groups, − 2.44 mm; 95% CI, − 3.88, − 1.00; *p* = 0.002).

**Conclusions:**

The use of either a slow or rapid activation protocol is effective in expanding the nasomaxillary complex, with greater expansion achieved in the anterior section of the nasal cavity using the slow rate. However, the expander design employed in the current study does not affect the dimensions of the retropalatal or retroglossal airways.

**Supplementary Information:**

The online version contains supplementary material available at 10.1186/s12903-022-02581-9.

## Background

Treatment of transverse maxillary constriction in adolescents usually involves skeletal expansion using tooth-supported or miniscrew-supported maxillary expanders [1]. Miniscrew-supported expanders were introduced to minimize unfavourable dento-alveolar effects and maximize skeletal effects [2]. Maxillary expansion can be either slow or rapid depending on the rate of jackscrew activation [3]. Recent research has shown that both slow maxillary expansion (SME) and rapid maxillary expansion (RME) using miniscrew-supported expanders are effective in correcting maxillary transverse constriction in adolescents, with SME offering the added benefit of less clinical complications [4], and better patient-reported outcomes [5].

In addition to the skeletal effect on the maxilla, miniscrew-supported rapid maxillary expansion has been shown to have a positive effect on the nasal width [6–8], airway volume [7, 9–12] and minimum cross sectional area of upper airway [7, 12]. The increase in airway dimensions was associated with decreased nasal airway resistance [7, 13], increased respiratory muscle strength [9] and increased nasal airflow [13]. Contrarily, other research found minimal effect of miniscrew-supported slow expansion on the airway [14]. One study [15] formerly compared the effect of different activation rates of miniscrew-supported expanders on the lateral dimensions of the nasal cavity, but not the dimensions of the retroglossal and retropalatal airways. None of the previous studies that evaluated the changes in the upper airway dimensions following miniscrew-supported maxillary expansion has compared the effects of the different activation rates. A recent systematic review that investigated the effects of miniscrew-supported expanders on the upper airway in adolescents has corroborated the lack of studies that compare the effects of different activation protocols on the upper airway dimensions [16]. Such a comparison, coupled with the results of previous research that demonstrated the advantages of SME over RME [4, 5], would allow the clinician to make an evidence-based decision regarding which activation protocol to use with miniscrew-supported expansion to benefit the patient.

Evaluation of the airway dimensions can be performed using lateral cephalometric radiographs [17]. Nonetheless, the two-dimensional representation of a three-dimensional structure may overlook important anatomic features [18]. Moreover, the superimposition of bilateral structures and the variation in head position may affect the accuracy of the measurements [19]. The use of medical computed tomography has been previously described to allow precise measurements of the airway [20]; however, its radiation dose is relatively high, and the scan is made in a supine position which affects the airway volume [21]. Cone-beam computed tomography (CBCT) currently offers an accurate method for visualization and assessment of the airway volume [18], with the patient seated in an upright position [22]. When compared to medical computed tomography, CBCT provides faster scanning speed, lower effective radiation dose and lower cost [23].

Hence, the purpose of this study was to retrospectively evaluate and compare the effects of slow and rapid miniscrew-supported maxillary expansion on the upper airway dimensions using CBCT. The null hypothesis was that there was no difference between rapid and slow activation protocols regarding their effects on the upper airway dimensions.

## Methods

Data of adolescent patients who had undergone miniscrew-supported maxillary expansion at the Department of Orthodontics, Faculty of Dentistry, Alexandria University until February 2022 were retrospectively collected. Approval was obtained from the university’s institutional review board (IORG:0008839, no. 0417–03/2022). The inclusion criteria included maxillary transverse deficiency treated using miniscrew-supported maxillary expansion, and availability of CBCT scans pre-expansion (T1) and 5 months post-expansion (T2) after removal of the appliance, with a field of view covering the whole upper airway. Maxillary transverse deficiency was quantified using dental casts by measuring the difference between the maxillary and mandibular widths. The maxillary width was measured between the right and left most concave points of the maxillary vestibule corresponding to the mesio-buccal cusp of the first molars. The mandibular width was measured between the right and left WALA ridge corresponding to the mesio-buccal groove of first molars) [24]. The exclusion criteria included history of orthodontic treatment, tonsillectomy, adenoidectomy or orthognathic surgery, craniofacial malformations, or syndromes.

**Fig. 1:**
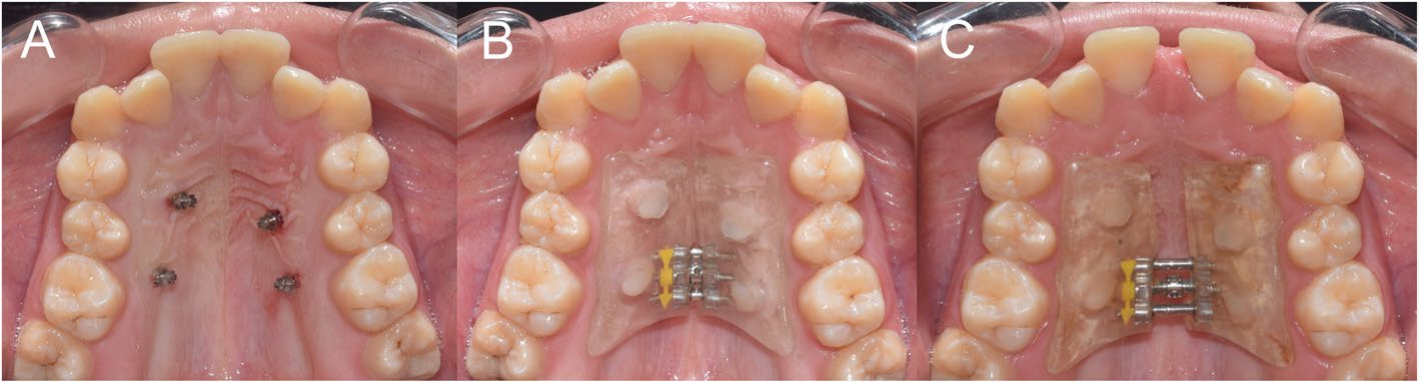
**A** Four palatal miniscrews; **B** Miniscrew-supported expander bonded to miniscrews using flowable composite; **C** The expansion screw activated

Oral assents and written informed consents were obtained from the patients and their parents, respectively, before treatment. All the patients were treated using a maxillary expander (Leone orthodontic products, Sesto Fiorentino, Firenze, Italy) supported on 4 palatal miniscrews (1.6 × 10 mm, Hubit Co Ltd., Ojeon-Dong, Korea), placed bilaterally between the first and second premolars and the first molars (Fig. 1). The sample was divided into two groups according to the activation protocol. The SME group activated the appliance once (0.2 mm) every other day, while the RME group activated the appliance twice (0.4 mm) daily until the transverse discrepancy was corrected. After correction, the appliance was left in place for retention. At the end of the retention period, 5 months after the initial activation, the appliance was removed, a CBCT scan was obtained, and fixed orthodontic treatment was started for each patient according to their respective treatment plan.

**Fig. 2:**
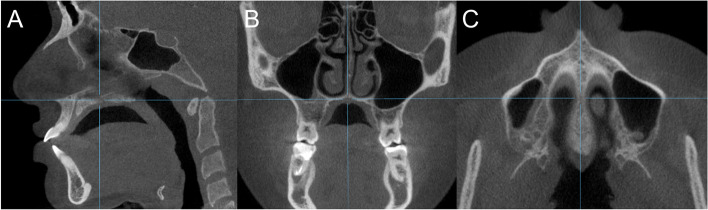
Orientation of the cone beam computed tomography scan in **A** Sagittal; **B** Coronal; and **C** Axial cuts

All CBCT scans were taken using i-CAT Next Generation device (Imaging Sciences International, Hatfield, Pa) with a 17 × 23 cm field of view, a 0.25-mm voxel size, a total scanning time of 25 seconds, 120 kVp and 5 mA. The patients were seated upright, with the head supported using a headrest, the Frankfurt Horizontal plane parallel to the floor, and the teeth in maximum intercuspation. The patients were instructed to breath naturally and not to swallow during the scanning procedure. CBCT scans were imported as Digital Imaging and Communications in Medicine files format into OnDemand3D™ software (Cybermed Inc., Seoul, Korea) and analysed using software measurement tools in the 3D module. The images were oriented so that the axial plane was parallel to the palatal plane in both the sagittal and coronal cuts, and the sagittal plane was parallel to the mid-palatal suture in the axial cut (Fig. 2).

**Fig. 3:**
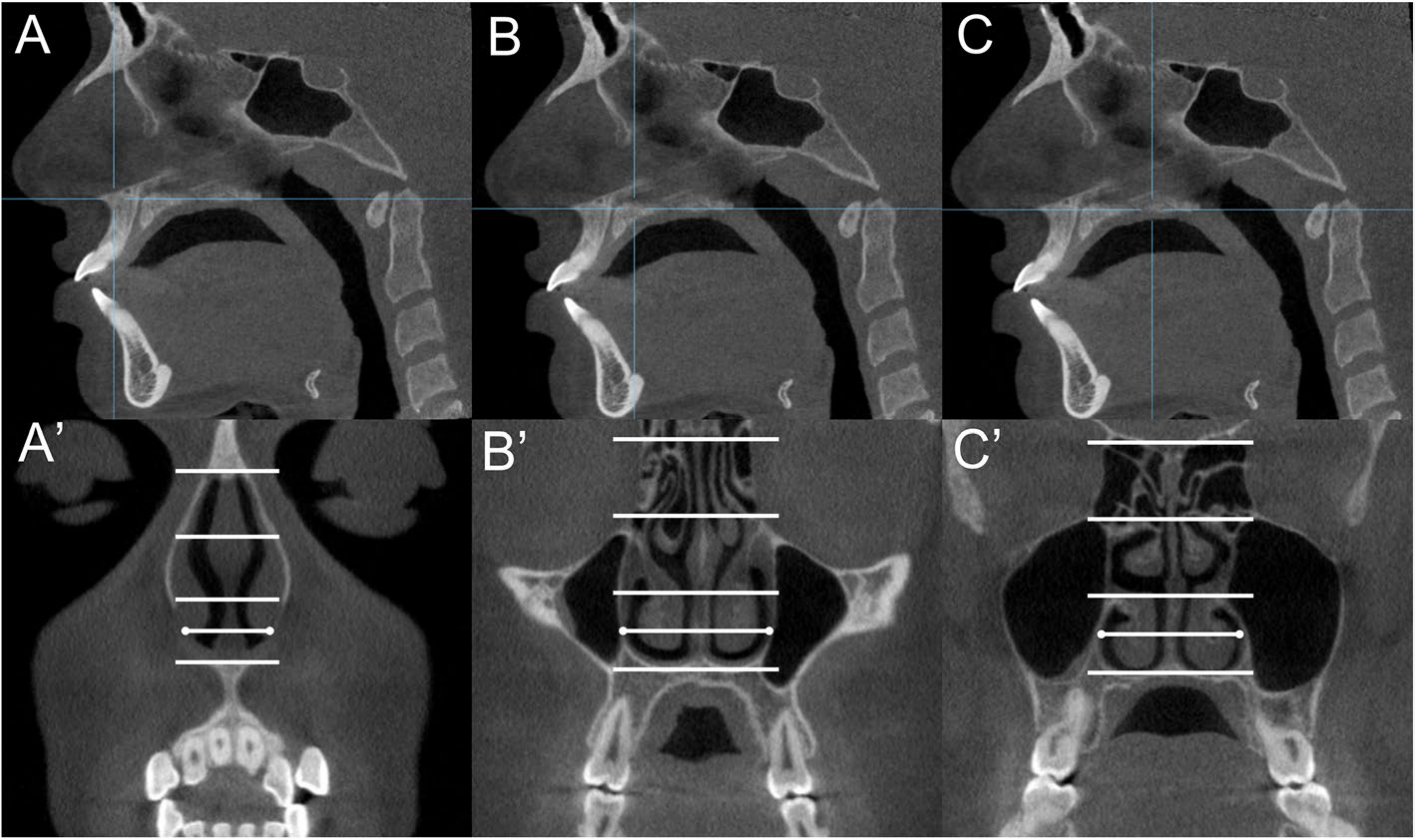
Measurement of the transverse dimension of the nasal cavity. **A**, **B**, and **C**, Sagittal cuts used to locate the anterior, middle, and posterior nasal cavity sections, respectively; **A**′, **B**′, and **C**′, The anterior, middle, and posterior coronal sections, respectively, used to measure the transverse dimension of the nasal cavity in the middle of the lower third of each section

The transverse dimensions of the anterior, middle, and posterior sections of the nasal cavity were measured on coronal cuts. The anterior section was located by dropping a perpendicular from Nasion point to the palatal plane in the sagittal cut (Fig. 3A). The middle (Fig. 3B) and posterior (Fig. 3C) sections were located 15 and 30 mm, respectively, posterior to the anterior section [25]. The transverse dimension was measured in the middle of the lower third of the nasal cavity on the anterior (Fig. 3A′), middle (Fig. 3B′) and posterior (Fig. 3C′) coronal sections.

**Fig. 4:**
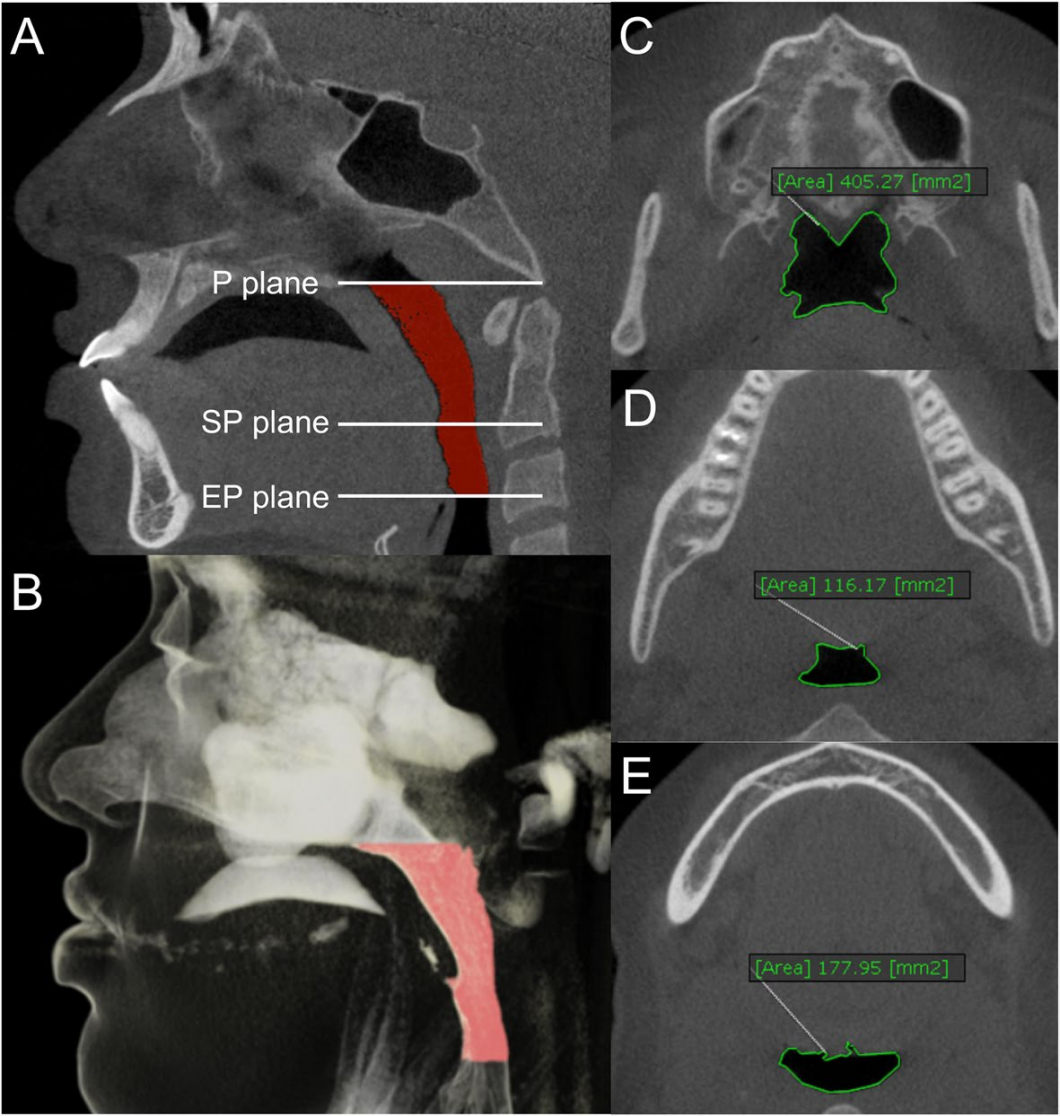
Measurement of the volume and cross-sectional area of upper airway. **A** Segmentation of the upper airway; **B** three-dimensional reconstruction of the upper airway; **C**, **D**, and **E**, Cross-sectional area of the airway at the P plane, SP plane and EP plane, respectively. (*P plane*: connecting posterior nasal spine to basion; *SP plane*: passing horizontally through the most postero-inferior point of soft palate; *EP plane*: passing horizontally through the most superior point of epiglottis)

The upper airway volume (Fig. 4) was defined as the volume between a superior “P plane” (connecting posterior nasal spine to basion) and an inferior “EP plane” (passing horizontally through the most superior point of epiglottis) [26]. The upper airway was further divided into retropalatal and retroglossal airways by the “SP plane” (passing horizontally through the most postero-inferior point of soft palate) [26]. After segmenting the airway using the software’s “Object Mask Tool”, its volume was measured in cubic millimetres using the “Threshold Tool”. The lower and upper threshold limits were determined for each CBCT scan using the “ROI” function which measures the minimum and maximum threshold values within a region of interest. The region of interest was the upper airway between the “P plane” and the “EP plane”. The cross-sectional area of the airway at the P plane, SP plane and EP plane was calculated using the software’s smart pen tool (Fig. 4).

**Fig. 5:**
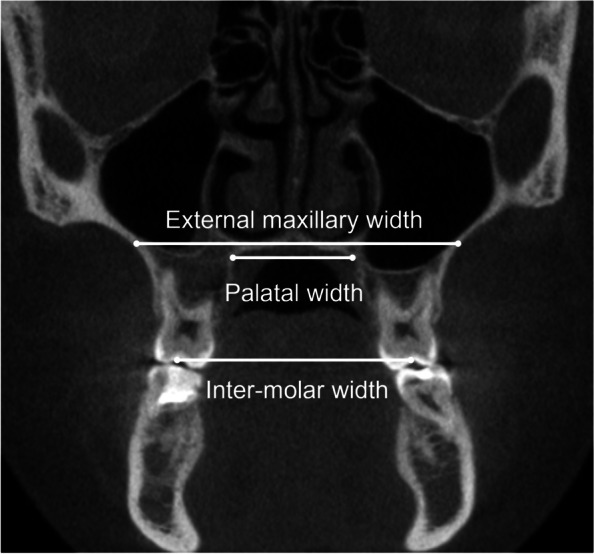
Measurement of dento-skeletal expansion

Dento-skeletal expansion was measured on the coronal slice passing through the furcation of maxillary first molars (Fig. 5). The maxillary inter-molar width was measured between the central fossae of the maxillary first molars, the external maxillary width was measured on a line connecting the depths of concavity of the lateral wall of maxillary sinuses, and the palatal width was measured on a line connecting the junction of the hard palate and lingual alveolar bone [12].

All CBCT measurements were performed by one author. Measurements were repeated after 2 weeks on 5 randomly selected datasets to assess intra-examiner reliability using intraclass correlation coefficient (ICC).

### Statistical analysis

The required sample size was based on Rosner’s method [27] and calculated using G*Power software (Version 3.1.9.4, Universität Düsseldorf, Germany) assuming 80% study power and 5% alpha error. As there were no previous studies comparing the dimensional changes in the upper airway following SME and RME using miniscrew-supported expanders, the sample size calculations were based on the results of a pilot study conducted on six patients. The calculated mean ± standard deviation (SD) total airway volume change of SME and RME was 10,996.09 mm^3^ ± 332.32 and 13,472.63 mm^3^ ± 2465.51, respectively. Based on comparison of means, using two-tailed test, the minimum required sample size was calculated (effect size = 1.40) to be 9 patients per group, and it was increased to 10 per group, so, the total required sample size was 20 patients [28]. The results of the pilot study were not included in the final analysis of the current study.

Normality was tested for all variables using descriptive statistics, plots (Q-Q plots and histogram), and Shapiro Wilk normality test. All variables showed normal distribution, so means and SD were calculated, and parametric tests were used. Comparisons between SME and RME were done using independent samples t-test with calculation of mean difference and 95% confidence intervals (CI). Comparisons between T1 and T2 within each group were done using paired samples t-test. Significance was set at *p* value < 0.05. Data were analysed using IBM SPSS for Windows, version 23.0 (IBM Corp., Armonk, NY).

## Results

The final analysis included 10 patients (8 females, 2 males, mean age: 14.26 ± 1.45 years) in SME group, and 10 patients (7 females, 3 males, mean age: 15.34 ± 1.16 years) in RME group. The mean amount of jackscrew expansion was 5.76 ± 0.84 mm and 5.80 ± 0.71 mm in SME and RME groups, respectively. The mean duration of expansion was 58.80 days (range 48 ~ 68 days) with SME, and 16.90 days (range 14 ~ 21 days) with RME.

Intra-examiner reliability for all the measured variables ranged from 0.935 to 0.999 indicating excellent reliability [29]. The data on intra-examiner reliability, including the 95% confidence intervals of the ICC for each variable, are provided in supplementary file 1.

Table 1 shows the changes in the airway dimensions from T1 to T2 within and between the SME and RME groups. Both groups showed a significant increase in the anterior, middle, and posterior nasal cavity width. However, SME showed a significantly greater increase of the anterior nasal cavity width than RME (Mean difference, 2.64 mm; 95% CI, 0.83, 4.45; *p* = 0.007).

**Table 1:** Changes in upper airway dimensions in SME and RME groups

	SME (*n* = 10)				RME (*n* = 10)				SME vs RME		
	T1	T2	T2-T1	*p* value^†^	T1	T2	T2-T1	*p* value^†^	Mean Difference	95% CI Lower, upper	*p* value^‡^
	Mean (SD)	Mean (SD)	Mean change (SD)		Mean (SD)	Mean (SD)	Mean change (SD)				
Nasal cavity width											
Anterior, mm	18.28 (3.10)	22.73 (2.60)	4.44 (1.98)	< 0.001*	18.03 (2.42)	19.83 (2.13)	1.80 (1.86)	0.01*	2.64	0.83, 4.45	0.007*
Middle, mm	28.10 (2.14)	31.10 (1.43)	3.00 (1.05)	< 0.001*	28.02 (3.81)	30.37 (3.23)	2.35 (1.11)	< 0.001*	0.65	−0.36, 1.67	0.19
Posterior, mm	28.89 (1.73)	30.89 (2.13)	1.99 (1.04)	< 0.001*	28.60 (3.71)	31.44 (2.85)	2.84 (1.31)	< 0.001*	−0.85	−1.96, 0.26	0.13
Retropalatal airway											
P-plane area, mm^2^	293.64 (113.54)	342.19 (141.94)	48.55 (117.27)	0.22	326.89 (114.08)	330.17 (115.09)	3.28 (77.75)	0.90	45.28	−48.20, 138.75	0.32
SP-plane area, mm^2^	247.98 (92.37)	228.10 (90.43)	−19.88 (39.23)	0.14	271.65 (122.23)	292.72 (126.04)	21.07 (103.01)	0.53	−40.95	−114.18, 32.29	0.26
Volume, mm^3^	6684.84 (2496.17)	6739.22 (2116.59)	54.38 (2501.06)	0.95	7408.10 (3464.20)	7818.52 (2837.69)	410.42 (1610.69)	0.44	− 356.04	− 2332.44, 1620.35	0.71
Retroglossal airway											
EP-plane area, mm^2^	241.89 (72.74)	237.26 (84.59)	−4.61 (62.34)	0.82	279.06 (112.41)	285.90 (107.75)	6.84 (102.23)	0.84	−11.46	−91.01, 68.10	0.77
Volume, mm^3^	3888.77 (1356.17)	4009.55 (1699.86)	120.78 (848.35)	0.66	5602.81 (3060.35)	5715.58 (2444.12)	112.77 (2971.78)	0.91	8.02	− 2045.22, 2061.25	0.99
Total airway volume											
Total volume, mm^3^	10,573.61 (3171.70)	10,748.77 (3158.31)	175.16 (2578.55)	0.84	13,010.91 (6305.14)	13,534.09 (4743.42)	523.19 (3634.39)	0.66	− 348.03	− 3308.59, 2612.54	0.81

*CI* Confidence interval, *SD* Standard deviation, *SME* Slow maxillary expansion, *RME* Rapid maxillary expansion, *T1* Pre-expansion, *T2* 5 months post-expansion

*Statistically significant at *p* value < 0.05

†Groups are compared using paired samples t-test

‡ Values are compared using independent samples t-test

The dimensions of the retropalatal and retroglossal airways did not change significantly from T1 to T2 in either group. No significant difference was found between the two groups.

Both SME and RME resulted in a significant increase of the maxillary width, palatal width, and inter-molar width (Table 2), with RME showing a significantly larger increase of inter-molar width than SME (Mean difference, − 2.44 mm; 95% CI, − 3.88, − 1.00; *p* = 0.002).

**Table 2:** Changes in maxillary measurements in SME and RME groups

	SME (*n* = 10)				RME (*n* = 10)				SME vs RME		
	T1	T2	T2-T1	*p* value^†^	T1	T2	T2-T1	*p* value^†^	Mean Difference	95% CI Lower, upper	*p* value^‡^
	Mean (SD)	Mean (SD)	Mean change (SD)		Mean (SD)	Mean (SD)	Mean change (SD)				
Inter-molar width, mm	41.83 (3.94)	46.32 (3.64)	4.49 (1.72)	< 0.001*	40.85 (3.33)	47.78 (2.89)	6.94 (1.32)	< 0.001*	−2.44	−3.88, −1.00	0.002*
External maxillary width, mm	59.10 (3.75)	62.34 (3.56)	3.24 (1.13)	< 0.001*	58.46 (1.96)	62.11 (1.97)	3.65 (1.65)	< 0.001*	−0.41	−1.74, 0.92	0.53
Palatal width, mm	23.05 (2.04)	27.71 (2.06)	4.66 (1.33)	< 0.001*	23.50 (4.24)	27.72 (3.96)	4.22 (1.87)	< 0.001*	0.44	−1.08, 1.97	0.55

*CI* Confidence interval, *SD* Standard deviation, *SME* Slow maxillary expansion, *RME* Rapid maxillary expansion, *T1* Pre-expansion, *T2* 5 months post-expansion

*Statistically significant at *p* value < 0.05

†Groups are compared using paired samples t-test

‡ Values are compared using independent samples t-test

## Discussion

The objective of the study was to compare the effect of slow and rapid activation rates of miniscrew-supported maxillary expanders on the upper airway dimensions using CBCT. Clinically, increasing the dimensions of the airway may alter the patients’ respiration [7, 9, 13]. Moreover, it may alleviate breathing disorders [30]. Linear parameters, cross-sectional area and volume of the airway were measured on CBCT to provide a complete evaluation of the upper airway [31]. The contrast between air and soft tissues allowed accurate segmentation and analysis of the airway dimensions using CBCT [18]. The lower and upper threshold limits of the airway were determined individually for each CBCT scan to measure the airway volume. Volume measurement using a constant threshold value was previously shown to result in more errors [18].

The results of the current study confirm the findings obtained previously that both SME and RME using bone-borne expanders produce a significant increase in maxillary width [4, 12]. However, RME resulted in significantly larger increase in inter-molar width than SME which may be attributed to the larger dental inclination that takes place with rapid activation compared to slow activation [4], possibly because of the rotation of the maxillary halves laterally [32]. Both SME and RME showed a significant increase in the palatal width in the current study. Similar results were previously obtained using rapidly activated miniscrew-supported expanders in adolescents [10, 12]. The increased transverse dimension of the palatal space possibly allows better tongue posture which in turn may have a positive effect on the oropharyngeal dimensions [33].

Both slow and rapid activation rates resulted in significant lateral expansion of the nasal cavity in addition to the maxillary expansion. A comparable result was previously obtained using slow [7], and rapid [6, 8] activation rates. The anatomical proximity of the maxillary and nasal bones accounts for the positive effect of the expander on the nasal cavity width [34]. To the contrary, other researchers reported smaller [35] or non-significant [36] change in the transverse dimensions of the nasal cavity following miniscrew-supported expansion. However, different landmarks were used to measure the nasal cavity width in the previous studies, hence the disparate results. In the current study, the increase in the anterior nasal cavity width was significantly larger with SME than RME. The change in the anterior nasal cavity dimensions is important because it is the area where the largest resistance to nasal airflow takes place, hence an increase in its dimensions may improve nasal breathing [37].

The effect of miniscrew-supported expansion on the upper airway volume was not significant regardless of the activation protocol used. A similar result was previously obtained by Kabalan et al. [14] using a slow miniscrew-supported expander. Likewise, Li et al. [7] found no significant change in the retropalatal and retroglossal airway volume following slow miniscrew-supported expansion, and Kavand et al. [10] reported a significant increase in the nasal cavity and nasopharyngeal volume, but not the oropharyngeal volume, following rapid miniscrew-supported expansion. Previous research investigating conventional tooth-supported expanders found that RME does not increase the oropharyngeal volume [26, 38]. The lack of a significant change may be attributed to the remoteness of the retropalatal and retroglossal airway from the point of force application. Ghoneima et al. [39] previously demonstrated that RME forces mainly affect the anterior craniofacial sutures. Hence, the increase in airway dimensions in the current study was limited to the nasal cavity.

Contrarily, other researchers reported a significant increase in the upper airway volume using rapid miniscrew-supported expansion [9, 12]. The disparate results between the studies could be accounted for by the differences in the methodology. Storto et al. [9] treated patients that were initially mouth-breathers, and some patients initially showed signs of airway constriction. Hence, expansion resulted in improvement of the airflow and, accordingly, the airway volume [9]. Mehta et al. [12] reported a significant increase in the oropharyngeal airway, however, their definition of the oropharyngeal airway did not include the retroglossal airway. Lanteri et al. [40] previously compared SME and RME using conventional tooth-supported appliances in children and found a significant increase in the nasopharyngeal volume following treatment. However, similar to the current study, no significant difference in the volumetric change was found between the two activation protocols [40].

No significant changes were found in the cross-sectional area of the airway in either group in the current study. The results obtained by Li et al. [7], using a slow miniscrew-supported expander, and Kim et al. [11], using a rapid miniscrew-supported expander, partially support the current findings. Both studies reported a significant increase in the cross-sectional area of the upper segment of the nasopharynx, but not the oropharynx [7, 11].

The conflicting findings between the studies may be related to the inconsistency in defining the borders of the airway [33], the variability in the CBCT scanning protocols [22], the variations in appliance design, and the difference in the amount of jackscrew expansion. Additionally, the different age ranges of the patients may have impacted the outcomes of the different studies because growth and development affect the dimensional changes of the airway [7, 12, 41]. Moreover, the difference in the time points of assessment may have caused the disparity between the different studies. Previous research [11] found no significant difference in nasopharyngeal volume immediately post-expansion, but found a significant increase 1 year post-expansion, possibly because the lapse of time allows soft tissue adaptation to the hard tissue changes produced by the expander. Hence, it is recommended to conduct a randomized clinical trial with long-term follow-up to confirm the results of this retrospective study.

Within the limitations of the current study, it is clinically recommended to employ a slow activation protocol when treating adolescent patients using the reported miniscrew-supported expander design. The slow rate successfully corrects the maxillary skeletal transverse discrepancy [4], with more nasal width expansion anteriorly, less clinical complications [4] and a better patient experience [5] than the rapid rate.

### Limitations

The CBCT scans were obtained in natural breathing which may have affected the airway volume because airway size and shape are affected by the respiration stage [26]. Another drawback is that no nasal airflow analysis was performed. The change in airway dimensions may affect the airway resistance and hence the airflow. In addition, during the CBCT assessment, the nasal cavity was not segmented and only the change in its transverse dimension was assessed. Segmentation was not performed because the tortuous pathways of the nasal cavity make defining its boundaries challenging [22]. It was not feasible to include an untreated control group in the current study based on ethical grounds. However, the CBCT records were obtained for both groups 5 months post-expansion, hence, any differences not pertaining to treatment were analogous in both groups. The short-term evaluation in the current study may be considered a limitation, therefore conducting another study with long-term follow-up is recommended. Another limitation is the relatively small sample size which might have an impact on the study power and the results’ interpretation, so further studies with larger sample size are needed. The data obtained in the current study could have been obtained using a lower radiation dose, however, the retrospective nature of the study precluded the use of CBCT with a lower radiation protocol. Hence, future research should aim to use the ALADA principle (As Low As Diagnostically Acceptable) when using CBCT and weigh potential benefits against possible risks [42].

## Conclusions

Based on the results of the current study comparing slow and rapid activation protocols of miniscrew-supported expanders, it can be concluded that:

1. The use of either activation protocol is effective in expanding the nasomaxillary complex, with greater expansion of the anterior section of the nasal cavity achieved using the slow activation protocol.
2. The miniscrew-supported expander design employed in the current study does not affect the dimensions of the retropalatal or retroglossal airways.

## Supplementary Information

**Additional file 1: Table 1.** Reliability analysis using ICC.

## Data Availability

The dataset used in the current research is available at synapse.org, under the title: Slow vs rapid miniscrew-supported maxillary expansion. DOI: 10.7303/syn36038127.1

## Abbreviations

ALADA: As Low As Diagnostically Acceptable
CBCT: Cone beam computed tomography
CI: Confidence intervals
ICC: Intraclass correlation coefficient
SD: Standard deviation
SME: Slow maxillary expansion
RME: Rapid maxillary expansion
