# Supplementary material for: Dimensional changes of upper airway after slow vs rapid miniscrew-supported maxillary expansion in adolescents: a cone-beam computed tomography study
Source: BMC Oral Health. 2022 Nov 24;22:529. doi: 10.1186/s12903-022-02581-9 (PMC9686034; doi:10.1186/s12903-022-02581-9)
Supplement: Supplementary file 1 — Additional file 1: Table 1. Reliability analysis using ICC. [file 12903_2022_2581_MOESM1_ESM.docx]

**Additional file 1.**

**Table 1**. Reliability analysis using ICC.

|  | **ICC (95% CI)** | **P value** |
| --- | --- | --- |
| **Nasal cavity width** | | |
| **Anterior** | 0.979 (0.914, 0.995) | <0.001* |
| **Middle** | 0.939 (0.754, 0.985) | <0.001* |
| **Posterior** | 0.971 (0.884, 0.993) | <0.001* |
| **Retropalatal airway** | | |
| **P-plane area** | 0.982 (0.929, 0.996) | <0.001* |
| **SP-plane area** | 0.999 (0.997, 0.999) | <0.001* |
| **Volume** | 0.985 (0.942, 0.996) | <0.001* |
| **Retroglossal airway** | | |
| **EP-plane area** | 0.998 (0.991, 0.999) | <0.001* |
| **Volume** | 0.999 (0.997, 0.999) | <0.001* |
| **Total airway volume** | | |
| **Total volume** | 0.995 (0.981, 0.999) | <0.001* |
| **Maxillary measurements** | | |
| **Intermolar width** | 0.995 (0.982, 0.999) | <0.001* |
| **External maxillary width** | 0.935 (0.739, 0.984) | <0.001* |
| **Palatal width** | 0.978 (0.910, 0.994) | <0.001* |

ICC: Intraclass Correlation Coefficient, CI: Confidence Interval

*Statistically significant at P value <0.05.
